# Direct Comparison of the Performance of Commonly Employed *In Vivo* F-actin Markers (Lifeact-YFP, YFP-mTn and YFP-FABD2) in Tobacco Pollen Tubes

**DOI:** 10.3389/fpls.2017.01349

**Published:** 2017-08-03

**Authors:** Adriana Montes-Rodriguez, Benedikt Kost

**Affiliations:** Cell Biology Division, Department of Biology, University of Erlangen-Nuremberg Erlangen, Germany

**Keywords:** *Nicotiana tabacum*, pollen tube, F-actin, talin, fimbrin, Abp140, Lifeact

## Abstract

*In vivo* markers for F-actin organization and dynamics are extensively used to investigate cellular functions of the actin cytoskeleton, which are essential for plant development and pathogen defense. The most widely employed markers are GFP variants fused to F-actin binding domains of mouse talin (GFP-mTn), Arabidopsis fimbrin1 (GFP-FABD2) or yeast Abp140 (Lifeact-GFP). Although numerous reports describing applications of one, or occasionally more, of these markers, are available in the literature, a direct quantitative comparison of the performance of all three markers at different expression levels has been missing. Here, we analyze F-actin organization and growth rate displayed by tobacco pollen tubes expressing YFP-mTn, YFP-FABD2 or Lifeact-YFP at different levels. Results obtained establish that: (1) all markers strongly affect F-actin organization and cell expansion at high expression levels, (2) YFP-mTn and Lifeact-YFP non-invasively label the same F-actin structures (longitudinally oriented filaments in the shank, a subapical fringe) at low expression levels, (3) Lifeact-YFP displays a somewhat lower potential to affect F-actin organization and cell expansion than YFP-mTn, and (4) YFP-FABD2 generally fails to label F-actin structures at the pollen tube tip and affects F-actin organization as well as cell expansion already at lowest expression levels. As pointed out in the discussion, these observations (1) are also meaningful for F-actin labeling in other cell types, which generally respond less sensitively to F-actin perturbation than pollen tubes, (2) help selecting suitable markers for future F-actin labeling experiments, and (3) support the assessment of a substantial amount of published data resulting from such experiments.

## Introduction

In plants, the actin cytoskeleton, which is composed of filamentous actin (F-actin), plays key roles in organelle transport (Wang and Hussey, 2015), membrane trafficking (Henty-Ridilla et al., 2013), cell division (Panteris, 2008), cell expansion (Smith and Oppenheimer, 2005), transport through plasmodesmata (White and Barton, 2011), gravity sensing (Blancaflor, 2013), programmed cell death (Smertenko and Franklin-Tong, 2011) and stomatal movements (Zhao et al., 2016). Consequently, F-actin has essential functions in plant development (Kost et al., 1999; Higaki et al., 2010; Zhu and Geisler, 2015) and pathogen defense (Porter and Day, 2016). Investigating and further characterizing these functions heavily depends on the visualization of F-actin organization and dynamics in different types of plant cells.

Excellent techniques are available to visualize F-actin organization in fixed plant cells either based on electron microscopy, or on fluorescence microscopy after staining with fluorescently labeled actin antibodies or derivatives of phalloidin, a membrane-permeable fungal metabolite that specifically associates with actin filaments (Wulf et al., 1979). Although these techniques have substantially contributed to our current understanding of the organization and function of the actin cytoskeleton in different plant cell types (Staiger et al., 2000), they do not allow observation of F-actin dynamics. For this purpose, during the past two decades a collection of markers and methods enabling F-actin imaging in living plant cells based on fluorescence microscopy have been developed and were extensively applied (Du and Ren, 2011).

Although other interesting markers for live-cell imaging of plant F-actin are available (e.g., Cheung et al., 2008; Wang et al., 2008), the most commonly used and widely accepted markers for this purpose (Du and Ren, 2011) are fluorescent fusion proteins composed of GFP (Green Fluorescent Protein) variants attached to F-actin binding domains of mouse talin (GFP-mTn; Kost et al., 1998), *Arabidopsis thaliana* fimbrin1 (GFP-FABD2; Sheahan et al., 2004; Voigt et al., 2005) or *Saccharomyces cerevisiae* Abp140 (Lifeact-GFP; Riedl et al., 2008; Era et al., 2009; Vidali et al., 2009). Although many reports describing applications of one or more of these three fusion proteins, as well as assessments of their performance as non-invasive F-actin markers, can be found in the literature (e.g., Sheahan et al., 2004; Wang et al., 2004, 2008; Voigt et al., 2005; Wilsen et al., 2006; van der Honing et al., 2011; Dyachok et al., 2014; Sparks et al., 2016), a direct comparison of all three markers, which is based on quantitative image analysis and takes expression level into account, has been missing.

Here, we present a direct comparison of F-actin labeling, as well as of effects on F-actin-dependent cell expansion, in transiently transformed tobacco pollen tubes expressing YFP-mTn, YFP-FABD2 or Lifeact-YFP fusion proteins at different levels. All fusion proteins tested contained the same yellow fluorescent protein (YFP), which was attached via a flexible 5× Glycine-Alanine linker to one of the three different F-actin binding domains introduced above. cDNAs coding for the different fusion proteins were cloned into the same plasmid vector and expressed under the control of the pollen specific Lat52 promoter (Twell et al., 1991) with an identical NOS polyA+ signal attached at the 3′ end.

For several reasons, transient transformation of tobacco pollen tubes is ideally suited to directly compare the performance of different fluorescent *in vivo* F-actin markers: (1) Application of this method results in F-actin marker expression in individual transformed pollen tubes at a wide range of different levels, which can be directly inferred from the brightness of fluorescence emission (Soboleski et al., 2005; Lo et al., 2015). This provides an optimal situation to comparatively analyze the ability of different fluorescent *in vivo* markers to non-invasively label F-actin at variable expression levels. (2) The rate of pollen tube growth is extremely sensitive to perturbation of the actin cytoskeleton (Gibbon et al., 1999; Vidali et al., 2001) and is expected to be reduced by the expression of fluorescent *in vivo* markers at levels that affect F-actin organization. Pollen tube growth at normal rates can therefore serve as an indicator of non-invasive labeling of *in vivo* F-actin organization by such markers. (3) F-actin organization in vegetative tobacco pollen tube cells has been extensively studied using a variety of techniques including electron microscopy (Lancelle and Hepler, 1992; Miller et al., 1996), immuno- or phalloidin labeling after fixation (Gibbon et al., 1999; Geitmann et al., 2000; Lovy-Wheeler et al., 2005; Wilsen et al., 2006) as well as live-cell imaging of fluorescent *in vivo* markers including GFP-mTn, GFP-FABD2 and Lifeact-GFP fusion proteins (Kost et al., 1998, 2000; Wilsen et al., 2006; Cheung et al., 2008; Vidali et al., 2009; Stephan et al., 2014).

Irrespective of the technique applied, longitudinally oriented F-actin fibers are invariably observed in the pollen tube shank, which appear to mediate myosin-dependent organelle transport (cytoplasmic streaming; Hepler et al., 2001). By contrast, F-actin organization at the pollen tube tip has remained controversial, as a subapical F-actin fringe (e.g., Kost et al., 1998; Lovy-Wheeler et al., 2005), as well as fine F-actin filaments in the cytoplasm directly underneath the apical plasma membrane (Lancelle and Hepler, 1992; Miller et al., 1996; Fu et al., 2001; Qu et al., 2013) were only detected based on the application of some of the established imaging techniques, and in some experiments using fluorescent *in vivo* markers. Interestingly, at different expression levels *in vivo* markers generally visualize distinct F-actin structures in pollen tubes. It is therefore essential to determine which F-actin structures are labeled by each of these markers at non-invasive expression levels at which F-actin organization is not affected.

With the study described here, we were pursuing the following key aims:

(1) Rank the performance of the three fluorescent fusion proteins tested (YFP-mTn, YFP-FABD2 and Lifeact-YFP) as non-invasive F-actin marker based on the following two criteria: (a) ability to reveal as much information as possible about F-actin organization in normally growing tobacco pollen tubes, and (b) potential to affect tobacco pollen tube F-actin organization and growth rate at high expression levels.

(2) Enhance our understanding of the organization of the pollen tube actin cytoskeleton through the identification of F-actin structures, which (a) can be non-invasively visualized in normally growing tobacco pollen tubes based on YFP-mTn, YFP-FABD2 or Lifeact-YFP expression, or (b) are only observed when one of these markers is expressed at levels that affect F-action organization and cell expansion.

Results obtained not only help deciding, which of the three markers tested should be preferentially employed for non-invasive F-actin labeling in future experiments, they are also important for the assessment of a large amount of data generated using these markers, which has already been reported in the literature (e.g., the paper by Kost et al. (1998) introducing GFP-mTn, the first GFP-based marker that was developed for F-Actin labeling in plants, has been cited more than 500 times to date). As pointed out in the discussion, although the study presented here focusses on tobacco pollen tubes, key insights gained are likely to be relevant for *in vivo* F-actin imaging also in other types of plant cells.

## Materials and Methods

### Plasmid Construction and Purification

Standard techniques (Sambrook and Russell, 2001), enzymes obtained from Fermentas (St. Leon-Rot, Germany: Phusion DNA polymerase) or New England Biolabs (Ipswich, MA, United States: all other enzymes), and oligonucleotides purchased from Eurofins MWG Operon (Ebersberg, Germany) or Metabion International AG (Planegg/Steinkirchen, Germany) were employed for recombinant DNA construction and analysis. PCR-amplified fragments as well as junctions between ligated fragments were confirmed by Sanger sequencing in all cases. Large-scale purification of plasmid DNA for particle coating and transient pollen transformation was performed using JetStar 2.0 Maxiprep Kits (Genomed GMBH; Löhne, Germany).

cDNA sequences were constructed coding for Lifeact-YFP, YFP-mTn and YFP-FABD2 fusion proteins composed of eYFP (AAX97736; BD Biosciences-Clontech; San Jose, CA, United States) fused via a flexible 5× Gly-Ala linker to different F-actin binding domains identical to those reported in the original publications introducing the three F-actin markers: (1) *S. cerevisiae* Abp140^1-17^ (AJT97542.1; Lifeact-YFP: Riedl et al., 2008), (2) *Mus musculus* Talin1^2345-2541^ (NM_011602.5; YFP-mTn: Kost et al., 1998) or (3) *A. thaliana* Fimbrin1^325-687^ (NM_001341826/AT4G26700; YFP-FABD2: Sheahan et al., 2004; Voigt et al., 2005). Also as described in the original reports (Kost et al., 1998; Sheahan et al., 2004; Voigt et al., 2005; Riedl et al., 2008), YFP is attached to C-terminus of the Abp140^1-17^ peptide in the Lifeact-YFP marker, and to the N-Terminus of the Talin1^2345-2541^ or the Fimbrin1^325-687^ domain in the YFP-mTn and YFP-FABD2 markers. The amino acid sequences of the peptides linking eYFP to the F-actin binding domain in the different markers are as follows (5× Gly-Ala linker underlined): PGGAGAGAGAGALEGT (Lifeact-YFP), SRGAGAGAGAGAG (YFP-mTn) and SRGAGAGAGAGAGK (YFP-FABD2).

The Lifeact-YFP, YFP-mTn and YFP-FABD2 cDNAs were inserted between the Lat52 promoter (Twell et al., 1991) and a NOS polyA+ signal (derived from pBI121; Jefferson et al., 1987) into a pUCAP (van Engelen et al., 1995) based vector (Kost et al., 1998). Resulting plasmids with a size of 4412 bp (pSLU29; Lifeact-YFP), 4854 bp (pWEN199; YFP-mTn) or 5349 bp (pSLU59; YFP-FABD2) were used for transient pollen transformation by particle bombardment.

### Plant Material

Tobacco (*Nicotiana tabacum* Petit Havana SR1) plants were grown at monthly intervals from seeds germinated in soil and maintained in a growth chamber under the following conditions: 16 h illumination (200–250 μmol m^-2^s^-1^) at 24°C and 8 h darkness at 18°C per day, relative humidity 60–65%. Fresh pollen collected from mature plants was used for transient transformation.

### Transient Transformation by Particle Bombardment

Fresh tobacco pollen was plated on solid culture medium (Read et al., 1993a,b) and bombarded with DNA-coated gold particles using a PDS 1000/he biolistic gun (Bio-Rad, Munich, Germany) as previously described (Kost et al., 1998; Johnson and Kost, 2010). Each batch of particles was coated with 4 μg plasmid DNA in the presence of 1 mg/ml protamine (Sun et al., 2015).

### Epifluorescence Microscopy, Expression Level Classification and Measurement of Pollen Tube Length

To acquire low magnification epifluorescence images, 6 h after gene transfer transiently transformed pollen tubes growing on solid culture medium were transferred onto a coverslip by cutting a section of the medium and flipping in upside-down directly on the glass surface as previously described (Kost et al., 1998; Johnson and Kost, 2010). Epifluorescence micrographs were recorded using an imaging workstation (Leica Microsystems; Wetzlar, Germany) based on an inverted epifluorescence microscope (DMI4000B) and equipped with an X-Cite^®^ 200DC light source, a YFP band-pass filter set (excitation: 490–510 nm, dichroic: LP 515 nm, emission: BP 520–550), a N PLAN 5×/0.12 lens and a peltier cooled b/w CCD camera (DFC365 FX), which was run in an 8-bit readout mode (dynamic range of 256 gray levels). All micrographs analyzed in this study were recorded using identical imaging parameters (e.g., camera exposure time: 1s).

**FIGURE 1:**
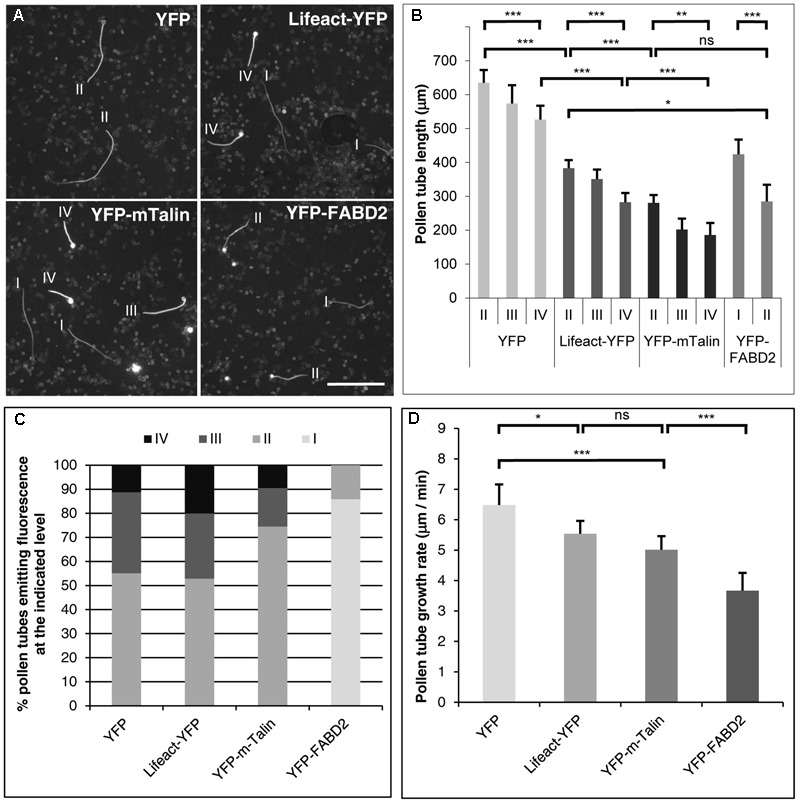
Effects of transient F-actin marker expression at different levels on tobacco pollen tube growth. **(A)** Microscopic epifluorescence images of fluorescent tobacco pollen tubes expressing YFP or the indicated F-actin marker at high levels under the control of the Lat52 promoter 6 h after gene transfer by particle bombardment. Images were recorded at low magnification (5× lens) using a CCD camera with a dynamic range of 256 gray levels keeping all imaging parameters constant (microscope/camera settings). I–IV: level of YFP or F-actin marker expression as defined in **(B)**. Scale bar: 500 μm. **(B)** Average length of pollen tubes expressing YFP or the indicated F-actin marker at different levels (I–IV), as determined on images recorded as described in **(A)**. Four different levels of YFP fluorescence emission were defined based on the gray level of the brightest pixels in the image of each analyzed pollen tube (excluding the pollen grain): level I (maximal gray level 1–129), level II (130–179), level III (180–199) and level IV (200–256). The average length of YFP, Lifeact-YFP or YFP-mTn expressing pollen tubes emitting bright fluorescence at levels II, III or IV was determined. By contrast, YFP-FABD2 expressing pollen tubes emitting fluorescence at levels I or II were analyzed, because none of these pollen tubes displayed brighter fluorescence. Error bars: 95% confidence interval. ANOVA (*post hoc* Bonferroni–Holm) test: ns, non-significant; ^∗^*P* < 0.05; ^∗∗^*p* < 0.01; ^∗∗∗^*p* < 0.001. Each data point represents 12–148 pollen tubes measured in four independent experiments [only few YFP-FABD2 expressing pollen tubes (*n* = 12) were found to emit fluorescence at level II]. **(C)** Chart indicating the percentage of all YFP, Lifeact-YFP or YFP-mTn expressing pollen tubes analyzed to generate the graph shown in **(B)**, which were emitting bright fluorescence at levels II, III or IV. Because none of the YFP-FABD2 expressing pollen tubes displayed fluorescence at levels III or IV, the percentage of these pollen tubes emitting fluorescence at levels I or II is displayed. **(D)** Average growth rate of morphologically unaffected pollen tubes expressing YFP or the indicated F-actin marker at lowest detectable levels as determined after confocal imaging of YFP fluorescence (**Figures 2–4**). Error bars: 95% confidence interval. ANOVA (*post hoc* Bonferroni–Holm) test: ns, non-significant; ^∗^*P* < 0.05; ^∗∗∗^*p* < 0.001. Each data point represents 20–24 pollen tubes measured in three independent experiments.

To broadly classify the level of YFP or YFP-fusion protein expression in brightly fluorescent pollen tubes, and to determine the length of these cells, micrographs were imported into IMAGEJ (National Institute of Health; Bethesda, MD, United States). Subsequently, the “Image/Adjust/Threshold” function was used to highlight in red color pixels displaying gray levels above an adjustable threshold. Moving this threshold stepwise down from the maximal gray level (256), the brightest pixels (the first ones to turn red) in the image of each pollen tube excluding the pollen grain was identified. It was necessary to exclude the pollen grain from this analysis, because pollen grains often contained parts of the living, transformed protoplast and in this case emitted very bright fluorescence as a consequence of their large diameter. Based on this procedure, transformed pollen tubes were broadly classified into four groups emitting fluorescence at four different levels (**Figure 1A**): level I (maximal gray level 1–129), level II (maximal gray level 130–179), level III (maximal gray level 180–199) and level IV (maximal gray level 200–256). To determine the length of analyzed pollen tubes, they were traced with the “freehand line” tool and measured (“Analyze/Measure”) after image calibration (“Analyze/Set Scale”).

### Confocal Microscopy and Measurement of Pollen Tube Growth Rate

To record confocal images of F-actin organization, 6–8 h after gene transfer transiently transformed pollen tubes were transferred onto a coverslip as described above for epifluorescence imaging. Serial confocal sections were imaged in the 6× line averaging mode at a step size of 0.5 μm using a TCS SP5 II laser scanning confocal microscope (Leica Microsystems; Wetzlar, Germany) and an HCX PL APO 63×/1.20 water immersion lens. Fluorescence was excited by a 514 nm argon laser and imaged in the range from 530 to 600 nm. Line-by-line sequential imaging was employed to quasi-simultaneously record confocal images of YFP-FABD2 labeled F-actin organization and transmitted light reference images (Differential Interference Contrast, DIC). Growth rates of individual pollen tubes were determined after confocal imaging of F-actin organization by measuring the distance between the positions of the apex on two images taken in the same focal plane at a 2 min interval.

### Statistical Analysis of Pollen Tube Length and Growth Rate Measurements

Microsoft Excel (Microsoft Corporation; Redmond, WA, United States) was employed to compute the average and the 95% confidence interval of individual sets of pollen tube length or growth rate measurements; as well as to assess the significance of differences between such data sets based on one-way ANOVA *post hoc* Bonferroni–Holm testing (performed using the XL Toolbox NG for Microsoft Excel^1^). *P*-values smaller than 0.05 indicate statistically significant differences between two data sets.

## Results

### All F-actin Markers Tested Reduce Pollen Tube Growth When Expressed at High-Level

A direct comparison was performed of effects of high-level expression of either free YFP, or of one of the F-actin markers Lifeact-YFP, YFP-mTn or YFP-FABD2, on the growth of tobacco pollen tubes transiently transformed by particle bombardment. In these experiments, the Lat52 promoter was used to drive transgene expression, which unlike the 35S promoter is highly active in pollen tubes (Twell et al., 1989, 1990, 1991; Sun et al., 2015). Presumably because gene transfer by particle bombardment results in the delivery of variable amounts of plasmid DNA to single cells, individual transformed pollen tubes displayed a wide range of expression levels (Johnson and Kost, 2010; Stephan et al., 2014; Sun et al., 2015). As fluorescence emission has been shown to be directly proportional to the expression level of fluorescent (fusion) proteins in living cells, quantitative fluorescence imaging can be employed to determine the expression level of such proteins in single cells (Soboleski et al., 2005; Lo et al., 2015). Six hours after gene transfer, a CCD camera with a dynamic range of 256 gray levels (8-bit) was employed to record low magnification (5× lens) epifluorescence micrographs of all transformed pollen tubes keeping all imaging parameters (microscope/camera settings) constant (**Figure 1A**). On these micrographs, brightly fluorescent pollen tubes strongly expressing transgenes were selected for length measurements. Based on the gray level of the brightest pixels in the image of each of the analyzed pollen tubes (excluding the pollen grain), they were broadly classified into four categories emitting YFP fluorescence at different levels: level I (maximal gray level 1–129), level II (maximal gray level 130–179), level III (maximal gray level 180–199) and level IV (maximal gray level 200–256) (**Figure 1A**).

Analysis of the average length of pollen tubes emitting fluorescence at different levels as defined above demonstrated that high-level expression of all marker proteins tested inhibited pollen tube growth in a dose-dependent manner (**Figure 1B**). Pollen tubes expressing free YFP were morphologically unaffected and displayed a length within the normal range (Cheung et al., 2008; Stephan et al., 2014; Sun et al., 2015), although at highest expression levels (level IV) pollen tube growth was weakly but statistically significantly reduced (**Figure 1B**). By contrast, all three F-actin markers strongly reduced pollen tube growth already at lower expression levels. At expression levels II, III and IV, Lifeact-YFP reduced pollen tube length by 39.7–55.5% as compared to free YFP expressed at level II. YFP-mTn expression at the same levels inhibited pollen tube growth slightly but detectably more strongly than Lifeact-YFP and cause a reduction of pollen tube length by 55.8–68.2%. Interestingly, YFP-FABD2 reduced pollen tube length to the same extent as YFP-mTn at expression level II, but was never found to be expressed at higher levels (levels III or IV). The low maximal expression level of YFP-FABD2 may be due to rapid turn-over, or to cytotoxicity that prevents marker accumulation to higher levels. Although all three F-actin markers strongly reduced pollen tube growth when expressed at high levels (**Figures 1A,B**), they only induced minor morphological alterations under these conditions, which included moderate tip swelling and a slightly irregular tube diameter (**Figures 1A, 5**).

In addition, the percentage of all YFP, Lifeact-YFP or YFP-mTn expressing pollen tubes analyzed to generate the graph shown in **Figure 1B**, which were emitting fluorescence at levels II, III or IV was determined (**Figure 1C**). This analysis further supported data shown in **Figure 1B**. In general, a decreasing percentage of pollen tubes expressing each marker emitted fluorescence at increasing levels (**Figure 1C**). Furthermore, a slightly larger proportion of Lifeact-YFP expressing pollen tubes, as compared to YFP-mTn expressing pollen tubes, emitted fluorescence at each of the two highest levels (III and IV), supporting the notion that Lifeact-YFP is a somewhat less invasive F-actin marker. Interestingly, YFP also appeared to be less frequently expressed at the highest level IV than Lifeact-YFP. Quantifying fluorescence emission based on maximal gray level is presumably responsible for this observation, as this will result in a moderate underestimation of the expression level of diffusely distributed YFP, as compared to F-actin markers that locally accumulate at cytoskeletal structures. Maximal YFP-FABD2 fluorescence at level II (see above) was only emitted by a small percentage of the pollen tubes expressing this marker, as determined when YFP-FABD2 expressing pollen tubes were included in the analysis, which emitted weaker fluorescence (level I) than any of the Lifeact-YFP and YFP-mTn expressing pollen tubes examined to generate **Figure 1B**.

Together, data shown in **Figures 1A–C** demonstrate that all three F-actin markers interfere with pollen tube growth when expressed at high levels. Furthermore, they indicate that at high expression levels Lifeact-YFP is somewhat more readily tolerated by living pollen tubes than YFP-mTn, whereas YFP-FABD2 appears to be considerably less stable and/or more cytotoxic than both these two markers.

### *In Vivo* Visualization of F-actin Organization in Pollen Tubes Expressing Different Markers at Minimal Detectable Levels

**FIGURE 2:**
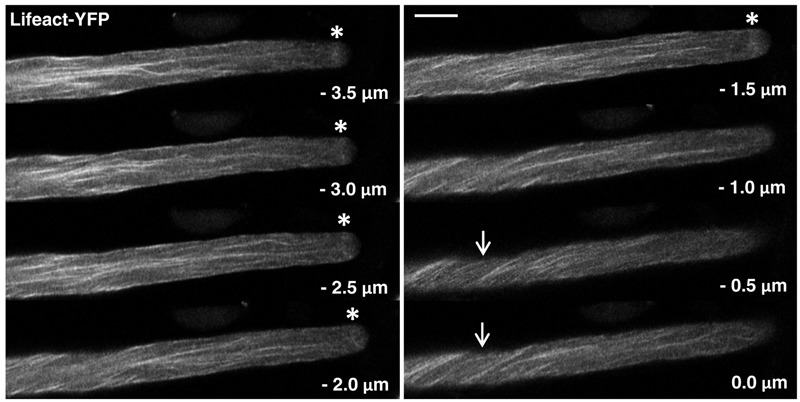
F-actin organization as visualized by confocal microscopy in normally growing tobacco pollen tubes expressing Lifeact-YFP at low level. Serial confocal sections taken at a step size of 0.5 μm (6× line averaging) are displayed. The pollen tube shown was growing at a rate of 6.2 μm/min after confocal imaging. 0.0 μm: first image of the series showing the cell cortex closest to the microscope lens. Arrows: helical arrangement of cortical F-actin; asterisks: subapical F-actin fringe. Scale bar: 10 μm.

**FIGURE 3:**
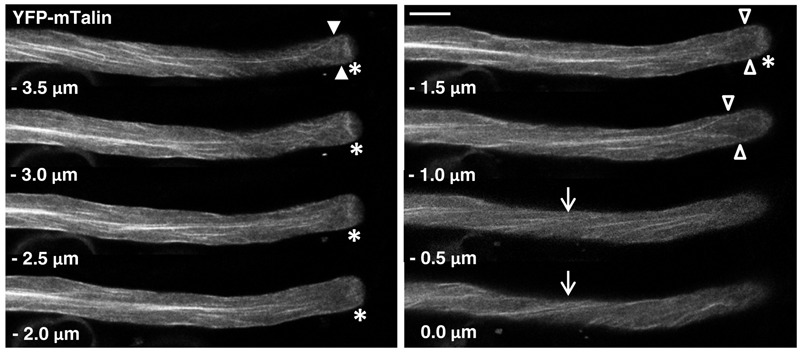
F-actin organization as visualized by confocal microscopy in normally growing tobacco pollen tubes expressing YFP-mTn at low level. Serial confocal sections taken at a step size of 0.5 μm (6× line averaging) are displayed. The pollen tube shown was growing at a rate of 5.5 μm/min after confocal imaging. 0.0 μm: first image of the series showing the cell cortex closest to the microscope lens. Arrows: helical arrangement of cortical F-actin; asterisks: subapical F-actin fringe; arrowheads (open and closed): actin fibers apparently extending from the longitudinally oriented F-actin network in the shank to the subapical F-actin fringe, which form a “fork-like subapical structure” connected to the fringe either directly (–3.5 μm; closed arrowheads) or indirectly (–1.0 μm; open arrowheads) via extensions in the extreme cortex (–1.5 μm; open arrowheads). Scale bar: 10 μm.

**FIGURE 4:**
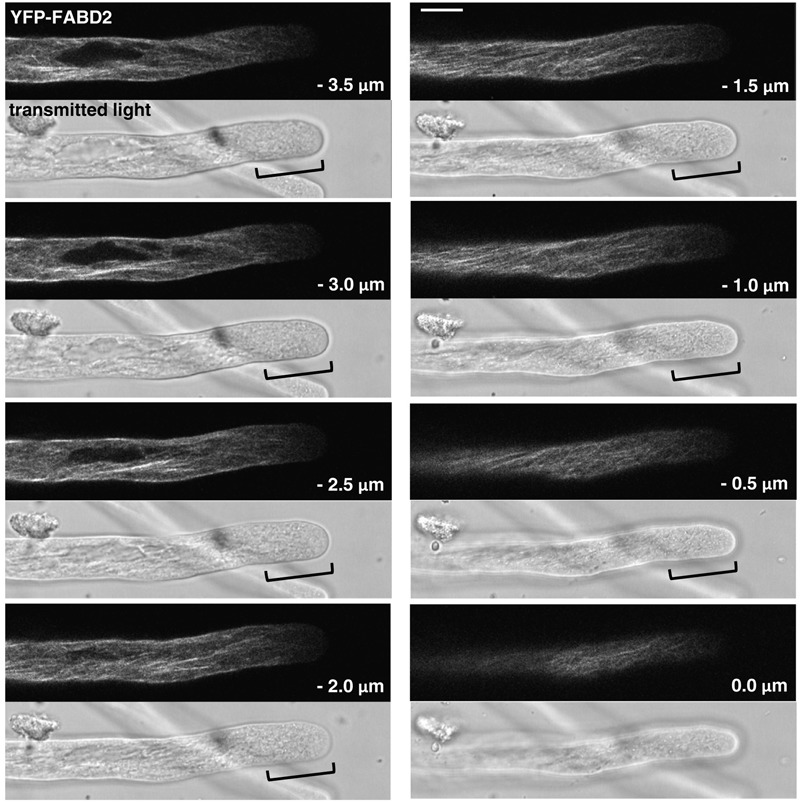
F-actin organization as visualized by confocal microscopy in tobacco pollen tubes expressing YFP-FABD2 at low level and growing at reduced rates. Serial confocal sections taken at a step size of 0.5 μm (6× line averaging) are displayed, each along with a simultaneously recorded transmitted light reference image (Differential Interference Contrast, DIC; displayed underneath the corresponding confocal section) to illustrate cell outline at the time of imaging. The pollen tube shown was growing at a rate of 3.66 μm/min after confocal imaging. 0.0 μm: first image of the series showing the cell cortex closest to the microscope lens. Brackets in reference images: region largely devoid of F-actin labeling covering the first ca. 15 μm at the tip. Scale bar: 10 μm.

Six to eight hours after gene transfer, F-actin organization was visualized using confocal microscopy in morphologically unaffected pollen tubes (regular diameter, normal cytoplasmic organization), which expressed the different F-actin markers at the lowest detectable levels and emitted the weakest fluorescence among all transformed pollen tubes observed (**Figures 2–4**). Analysis of the growth rates of all imaged pollen tubes showed that Lifeact-YFP and YFP-mTn expressing pollen tubes were elongating nearly as rapidly as weakly fluorescent YFP expressing pollen tubes at average rates of more than 5 μm/min (**Figure 1D**), which are within the range of normal growth rates of cultured tobacco pollen tubes reported in the literature (Cheung et al., 2008; Stephan et al., 2014; Sun et al., 2015). By contrast, YFP-FABD2 expressing pollen tubes were growing significantly more slowly. These results demonstrate that Lifeact-YFP and YFP-mTn allow F-actin visualization in normally elongating pollen tubes. They also further support the notion that Lifeact-YFP has a slightly lower potential to affect pollen tube growth than YFP-mTn, and indicate that YFP-FABD2 interferes with this process substantially more strongly than either of the other two markers. These observations are perfectly consistent with the quantification of effects of high-level expression of the different F-markers on pollen tube growth (**Figures 1A–C**).

Serial confocal optical sectioning showed that YFP was diffusely distributed throughout the pollen tube cytoplasm (Supplementary Figure S1), as reported in the literature (Helling et al., 2006; Klahre et al., 2006; Sun et al., 2015). Lifeact-YFP (**Figure 2**) and YFP-mTn (**Figure 3**) labeling patterns in normally elongating pollen tubes were nearly indistinguishable and revealed the same F-actin organization as previously reported in the literature (Kost et al., 1998, 2000; Gibbon et al., 1999; Geitmann et al., 2000; Lovy-Wheeler et al., 2005; Wilsen et al., 2006; Cheung et al., 2008; Vidali et al., 2009; Rounds et al., 2014; Stephan et al., 2014). Fine, primarily longitudinally oriented actin fibers where observed in the pollen tube shank, which as judged based on differences in their brightness appeared to represent bundles of variable numbers of actin filaments. In the cell cortex, these actin fibers generally displayed a helical arrangement (arrows). Furthermore, a subapical F-actin fringe (asterisks) was observed about 5 μm away from the extreme apex at the interface between the regular cytoplasm and the apical clear zone, from which all organelles apart from small transport vesicles are excluded (Hepler et al., 2001). Neither Lifeact-YFP nor YFP-mTn labeling allowed conclusions with regards to the arrangement or orientation of individual actin filaments within this sub-apical F-actin fringe. Interestingly, as previously reported (Kost et al., 1998; Geitmann et al., 2000; Lovy-Wheeler et al., 2005; Vidali et al., 2009; Rounds et al., 2014), actin fibers were frequently observed (17 or 19% of Lifeact-YFP or YFP-mTn expressing pollen tubes, respectively) to extend from the longitudinally oriented F-actin network in the shank to the F-actin fringe in the subapical cell cortex (**Figure 3**, arrowheads), indicating possible direct connections between these F-actin structures. Filamentous F-actin structures in the apical clear zone between the extreme apex and the subapical F-actin fringe could not be clearly detected in any of the analyzed normally elongating Lifeact-YFP or YFP-mTn expressing pollen tubes. Despite the essentially identical labeling patterns obtained with Lifeact-YFP and YFP-mTn, but consistent with the slightly lower potential of Lifeact-YFP to affect pollen tube growth (**Figure 1**), F-actin imaging in normally elongating tobacco pollen tubes appeared to be somewhat more effective with this marker. Presumably, a slightly larger proportion of all transformed pollen tubes displayed non-invasive F-actin labeling when Lifeact-YFP was expressed. Furthermore, cytoplasmic background labeling and actin filament bundling in the shank appeared to be a little less pronounced in pollen tubes expressing Lifeact-YFP. By contrast, YFP-mTn expression seemed to visualize the sub-apical F-actin fringe somewhat more clearly. However, none these minor differences were sufficiently substantial to allow confirmation by quantitative analysis.

F-actin organization as observed by confocal imaging of pollen tubes expressing YFP-FABD2 at lowest detectable levels (**Figure 4**), which were elongating at reduced rates (**Figure 1D**), substantially differed from the Lifeact-YFP and YFP-mTn labeling patterns in normally growing pollen tubes described above (**Figures 2, 3**). Although the shank of YFP-FABD2 expressing pollen tubes also contained primarily longitudinally oriented actin fibers, these fibers generally appeared to form a more fuzzily labeled and less regularly arranged network, suggesting that YFP-FABD2 may induce F-actin reorganization in the pollen tube shank already at minimal detectable expression levels. Most strikingly, in none of the pollen tubes expressing YFP-FABD2 a subapical F-actin fringe or actin fibers extending from the longitudinally oriented F-actin network in the shank to the subapical cell cortex were observed, suggesting that these F-actin structures are either disrupted or not labeled by this marker. In fact, almost no F-actin labeling was detectable in a region covering the first 15–30 μm at the tip of YFP-FABD2 expressing pollen tubes.

### All Markers Tested Affect Pollen Tube F-actin Organization When Expressed at High Levels

**FIGURE 5:**
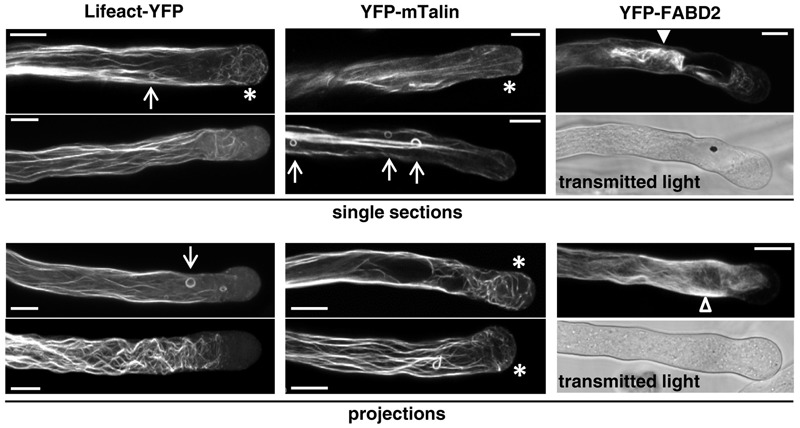
F-actin organization as visualized by confocal microscopy in tobacco pollen tubes expressing the indicated F-actin markers at high levels. Single sections **(Upper)** or projections of serial sections **(Lower)** through representative pollen tubes expressing the indicated F-actin marker at high level are displayed. Transmitted light reference images (Differential Interference Contrast, DIC; medial plane) are displayed underneath fluorescence micrographs of YFP-FABD2 expressing pollen tubes. All pollen tubes shown had completely stopped growing when F-actin organization was imaged. Arrows: F-actin rings; asterisks: fine F-actin fibers extending into the apical cytoplasm; arrowhead (closed): F-actin aggregate; arrowhead (open): diffusely labeled cytoplasm. Scale bars: 10 μm.

Confocal imaging of brightly fluorescent pollen tubes displaying morphological aberrations (irregular diameter, disturbed cytoplasmic organization), which expressed Lifeact-YFP or YFP-mTn at high levels that inhibited cell expansion (**Figures 1A–C**), demonstrated that under these conditions each of the two markers induced massive F-actin bundling and reorganization (**Figure 5**). In the shank of all imaged pollen tubes a dense network of brightly labeled, thick F-actin fibers was observed, which appeared to be more curved and less regularly arranged than the finer F-actin fibers in the shank of normally elongating pollen tubes expressing Lifeact-YFP or YFP-mTn at minimal levels (**Figures 2, 3**). Furthermore, small F-actin rings with a diameter of a few micrometers were often observed in the shank of pollen tubes expressing Lifeact-YFP or YFP-mTn at high-levels (**Figure 5**, arrows). Interestingly, in most of these pollen tubes neither a subapical F-actin fringe nor actin fibers connecting the subapical cell cortex to the F-actin network in the shank were visible, whereas fine F-actin fibers were often found to extend into the apical cytoplasm all the way to the extreme tip (**Figure 5**, asterisks). Like the small F-actin rings observed in the shank, these fine F-actin fibers in the in apical cytoplasm clearly need to be considered artifacts induced by high-level Lifeact-YFP or YFP-mTn expression, as neither of these two structures was ever observed in normally growing pollen tubes expressing one of the two markers at minimal levels (**Figures 2, 3**).

As discussed above, YFP-FABD2 interferes with the growth of pollen tubes already at minimal detectable expression levels (**Figure 1D**), and reaches substantially lower maximal expression levels than Lifeact-YFP or YFP-mTn in these cells (**Figures 1A–C**). Comparing F-actin organization in pollen tubes expressing YFP-FABD2 at minimal detectable (**Figure 4**) or high (**Figure 5**) levels indicates that YFP-FABD2, like Lifeact-YFP and YFP-mTn, in a dose-dependent manner can stimulate F-actin bundling and the formation of thick actin fibers in the pollen tube shank. However, in addition to labeling these fibers highly expressed YFP-FABD2 generally also massively accumulated in diffusely labeled cytoplasmic regions, indicating local F-actin disruption (**Figure 5**, projections: open arrowhead). At maximal expression levels, YFP-FABD2 labeled F-actin structures frequently collapsed into one large aggregate near the pollen tube tip (**Figure 5**, single sections: closed arrowhead). Interestingly, irrespectively of expression level YFP-FABD2 failed to label any of the F-actin structures that can be visualized at the tip of pollen tubes expressing Lifeact-YFP or YFP-mTn either at low (subapical F-actin fringe, actin fibers connecting this structure to the F-actin network in the shank) or high (fine F-actin fibers extending into the in apical cytoplasm) levels.

## Discussion

### F-actin Structures That Can Be Non-invasively Visualized in Normally Growing Tobacco Pollen Tubes by *In Vivo* Markers Tested in This Study

When expressed at low levels, Lifeact-YFP and YFP-mTn consistently label the same F-actin structures in tobacco pollen tubes: (a) a network of primarily longitudinally oriented fine actin fibers in the shank, which are helically arranged in the cell cortex, and (b) a subapical F-actin fringe (**Figures 2, 3**). In addition, under the same conditions actin fibers connecting these two structures, which extend from the subapical cell cortex into the shank, are often visualized by each of the two markers (**Figure 3**). Together, these structures appear to constitute the tobacco pollen tube actin cytoskeleton as it can be non-invasively visualized using *in vivo* markers tested in this study at normal growth rates at which F-actin organization is unlikely to be substantially affected. The same structures were already described in the original reports establishing YFP-mTn as an *in vivo* F-actin marker (Kost et al., 1998, 2000), were more recently also imaged in living tobacco and lily pollen tubes expressing a Lifeact-mEGFP construct (Vidali et al., 2009; Rounds et al., 2014) and, in addition, can be observed based on optimized fixation and immuno- or phalloidin labeling procedures in tobacco, lily, poppy and maize pollen tubes (Gibbon et al., 1999; Geitmann et al., 2000; Lovy-Wheeler et al., 2005; Wilsen et al., 2006). As these structures can be visualized using different techniques in pollen tubes of several species, they are likely to represent key components of the actin cytoskeleton required for the tip growth of these cells. The different techniques available for observation of these structures have distinct specific advantages: whereas labeling with Lifeact-YFP, YFP-mTn or similar markers allows investigation of F-actin dynamics *in vivo*, staining of the sub-apical F-actin fringe in fixed pollen tubes revealed details that are not resolved by the two *in vivo* markers, and indicate that this structure is composed of a parallel array of longitudinally oriented short actin fibers (Gibbon et al., 1999; Lovy-Wheeler et al., 2005; Wilsen et al., 2006). New tools and methods need to be developed to determine whether the actin fringe in normally growing pollen tubes is also composed of similar arrays of short longitudinal actin fibers.

In addition to the F-actin structures described in the previous paragraph, which are commonly observed in pollen tubes of different species using *in vivo* markers expressed at low levels, or based on optimized immuno- or phalloidin labeling, actin fibers occasionally were also reported to be present in the cytoplasm at the extreme apex of tobacco (Fu et al., 2001) and Arabidopsis (Qu et al., 2013) pollen tubes expressing GFP-mTn or Lifeact-GFP markers, respectively, or of lily pollen tubes analyzed by electron microscopy (Lancelle and Hepler, 1992; Miller et al., 1996). As suggested in the literature (Fu et al., 2001; Kost, 2008; Qu et al., 2013, 2015; Stephan et al., 2014), actin fibers in the apical cytoplasm may serve as tracks for active myosin-mediated transport of secretory vesicles to the plasma membrane that possibly is required for pollen tube tip growth. However, actin fibers in the cytoplasm between the subapical F-actin fringe and the apical plasma membrane could not be clearly detected based on optimized immuno- or phalloidin labeling in tobacco, lily, poppy or maize pollen tubes (Gibbon et al., 1999; Geitmann et al., 2000; Lovy-Wheeler et al., 2005; Wilsen et al., 2006), and were never observed in normally elongating tobacco pollen tubes expressing YFP-mTn or Lifeact-YFP at low levels in the study presented here. This indicates that such fibers at least in tobacco pollen tubes can’t be non-invasively labeled by YFP-mTn or Lifeact-YFP, and may be too thin to be detectable by currently available techniques other than electron microscopy. Images of actin fibers in the apical cytoplasm of tobacco pollen tubes labeled by an “enhanced” GFP-mTn marker, which were presented in a previous report (Fu et al., 2001), presumably show pollen tubes that expressed this marker at high levels at which F-actin organization was affected. These images closely resemble our images of tobacco pollen tubes expressing YFP-mTn or Lifeact-YFP at high levels (**Figure 5**), in which no subapical F-actin fringe, but brightly labeled, thick F-actin fibers in the shank as well as fine F-actin fibers extending into the apical cytoplasm are visible. Information concerning the growth rates of imaged pollen tubes expressing “enhanced” GFP-mTn, or the level of expression of this marker in these cells, has not been reported (Fu et al., 2001). The “enhanced” GFP-mTn marker (GFP^S65C^; Reichel et al., 1996) used in the previous study (Fu et al., 2001), “regular” GFP-mTn (GFP^F64L,S65T^; Kost et al., 1998, 2000) and the YFP-mTn fusion protein (GFP^S65G,V 68L,S72A,T203Y^) we employed for the work presented here are not expected to perform substantially differently (Cubitt et al., 1995; Heim et al., 1995; Cormack et al., 1996; Ormo et al., 1996). In fact, when “enhanced” GFP-mTn (Fu et al., 2001) and “regular” GFP-mTn (Kost et al., 1998) were directly compared in tobacco pollen tubes, no differences in signal quality or labeling pattern were observed (Wilsen et al., 2006).

More recently, the dynamic behavior of Lifeact-GFP labeled actin fibers was investigated at the extreme apex of growing Arabidopsis pollen tubes (Qu et al., 2013). A subapical F-actin fringe was not clearly visible in the analyzed pollen tubes, and neither their growth rates nor the level at which they expressed Lifeact-GFP have been reported. Considering data presented here, further analysis appears to be required to determine whether the actin cytoskeleton was perhaps affected by high-level Lifeact-GFP expression in the analyzed Arabidopsis pollen tubes, or may be differently organized in these cells as compared to pollen tubes of other species including tobacco, lily, poppy and maize.

### Important Considerations When Using Markers Tested in This Study for F-actin Visualization in Pollen Tubes and Other Cells

As discussed above, at low expression levels Lifeact-YFP and YFP-mTn non-invasively and detectably label the same F-actin structures in normally growing tobacco pollen tubes, which can also be observed using other techniques in pollen tubes of different species. This establishes that both markers are excellent tools to investigate pollen tube F-actin structures and dynamics *in vivo*. Although this was not investigated in the context of the study presented here, both markers have also been shown to allow monitoring of effects of drug treatments on F-actin organization (e.g., Vidali et al., 2009; Stephan et al., 2014).

To visualize the actin cytoskeleton, *in vivo* F-actin markers can readily be expressed in pollen tubes either transiently after particle bombardment (Kost et al., 1998; this study) or stably after genetic transformation (Stephan et al., 2014). Transient expression generates results within a few hours after gene transfer, but produces pollen tubes that express F-actin markers at highly variable levels. Stable expression can only be analyzed at least one generation time after gene transfer, but has the advantage that all pollen tubes derived from plants, which are homozygous for inserted transgenes coding for F-actin markers, generally express these markers at equal levels. Irrespective of the transformation method used, analysis of unaffected F-actin organization requires imaging of pollen tubes that are growing at normal rates. To exclude effects on the observed F-actin organization not only of marker overexpression but also of photo toxicity, we routinely disregard all images of pollen tubes, which are not continuing to grow normally after imaging.

Our results demonstrate that Lifeact-YFP clearly is the marker of choice for F-actin visualization in tobacco pollen tubes, as it has a somewhat lower tendency than YFP-mTn to affect actin organization, and strictly F-actin-dependent cell expansion, when expressed in these cells. However, YFP-mTn works almost equally well and results obtained with this marker are certainly perfectly valid, if care is taken to ensure that only normally growing pollen tubes are analyzed. Interestingly, YFP-mTn appears to label the subapical F-actin fringe slightly more effectively than Lifeact-YFP. Stronger interaction with this F-actin structure, which appears to be absolutely essential for cell expansion (Gibbon et al., 1999; Vidali et al., 2001; Stephan et al., 2014), may be responsible at least in part for the somewhat higher potential of YFP-mTn to interfere with this process.

As it is the case for most, if not all, GFP-based makers, when expressed above a marker specific threshold level Lifeact-YFP and YFP-mTn affect their target structure and therefore stop functioning as non-invasive markers. Because pollen tube growth is extremely sensitive to perturbation of the actin cytoskeleton, the growth rate of these cells can be seen as a suitable indicator of the threshold expression level, above which *in vivo* markers start to substantially affect F-actin organization. In normally growing pollen tubes, F-actin markers are likely to be expressed below this threshold. Interestingly, a recent report indicated that in transgenic Arabidopsis plants stably expressing a fluorescent Lifeact-Venus fusion protein at relatively high levels root hair tip growth was not significantly inhibited, although the rate of dynamic reorganization, as well as of drug-induced depolarization, of the actin cytoskeleton in root epidermal cells was clearly reduced (van der Honing et al., 2011). By contrast, at minimal detectable levels of Lifeact-Venus expression, neither tip growth nor actin dynamics were reported to be affected. In case Lifeact-Venus was in fact expressed at the same level in the analyzed root epidermal cells and root hairs, these observations indicate that root hair tip growth may tolerate some interference with F-actin dynamics caused by Lifeact-Venus expression at elevated levels. It is important to note in this context that compared to root hairs pollen tubes display a much higher growth rate (at least ca. 5×; Kost, 2008), which is likely to be substantially more sensitive to reduced F-actin dynamics. This assumption is consistent with the observation that pollen tube tip growth is exceptionally sensitive to F-actin disrupting drugs (Gibbon et al., 1999; Vidali et al., 2001).

In most cells types other that pollen tubes, or when structures with less critical functions than the pollen tube actin cytoskeleton are observed, it clearly is more difficult to define an expression level threshold for non-invasive labeling by GFP-based markers. In such cases, it appears advisable to image cells that express GFP-based markers for F-actin or other structures at the lowest detectable expression level, possibly under the control of promoters whose activities have been selected for best performance (Dyachok et al., 2014), or are inducible and can be titrated to suitable levels (Finka et al., 2007). For optimal *in vivo* visualization of the actin cytoskeleton or other structures, it is of course also important to employ the best available fluorescent proteins, which display the brightest fluorescence and the lowest tendency to self-associate (Dyachok et al., 2014). Replacing regular YFP by monomeric YFP (mYFP^A207K^), which shows a somewhat reduced self-association tendency (Zacharias et al., 2002), or by monomeric forms of proteins reported to emit even brighter fluorescence (e.g., mWASABI; Ai et al., 2008), may further enhance the performance of the F-actin markers tested in this study. However, this appears unlikely to result in major improvements, since YFP already is among the brightest fluorescent proteins available and only weakly interacts with itself (Ormo et al., 1996; Zacharias et al., 2002). Consistent with this assumption, not only high-level Lifeact-YFP expression in pollen tubes (this study), but also strong expression of a monomeric Lifeact-mEGFP fusion protein in moss protonemal cells has been shown to interfere with tip growth (Vidali et al., 2009).

At high expression levels, at which pollen tube growth rates are reduced, both Lifeact-YFP and YFP-mTn induced actin bundling and reorganization. Whereas a subapical actin fringe was often not detected under these conditions, cytoplasmic actin fibers generally appeared to be thicker, more abundant as well as more randomly oriented, and frequently extended from the shank all the way to the extreme apex. As discussed above, we believe F-actin structures exclusively visible in tobacco pollen tubes expressing Lifeact-YFP or YFP-mTn at levels at which tip growth is inhibited have to be considered structural artifacts. Interestingly, tobacco pollen tubes growing at reduced rates because of high-level Lifeact-YFP or YFP-mTn expression often contained small cytoplasmic F-actin rings. Similar F-actin rings have previously been observed in different types of plants cells, which expressed GFP-based F-actin markers (including Lifeact-YFP and YFP-mTn) or were labeled with phalloidin-derived fluorescent probes (Frost and Roberts, 1996; Kost et al., 1998; Wilsen et al., 2006; Chaidee et al., 2008; Smertenko et al., 2010). Like GFP-based markers, at elevated concentrations these probes potentially stabilize F-actin (Cooper, 1987). Whether small F-actin rings observed in cells types other than pollen tubes are components of the normal actin cytoskeleton of these cells, or perhaps were also formed as a consequence of F-actin labeling procedures, remains to be further investigated. Whereas the potential of YFP-mTn to induce F-actin bundling and reorganization, as well as to inhibit cell expansion, when expressed at high-levels in plant cells has been well documented in the literature (Kost et al., 1998, 2000; Ketelaar et al., 2004; Wilsen et al., 2006), to date Lifeact-YFP was generally described to have no, or a much lower, potential to induce such effects (Smertenko et al., 2010; Du and Ren, 2011; van der Honing et al., 2011; Qu et al., 2013, 2015). By contrast, data presented here establish that Lifeact-YFP affects F-actin organization and cell growth in a similar manner as YFP-mTn at expression levels that are only somewhat higher. Consistent with this observation, as indicated above a Lifeact-mEGFP fusion protein also inhibits tip growth when expressed at high levels in moss (Vidali et al., 2009). Consequently, also when fluorescent Lifeact-fusion proteins are employed as *in vivo* F-actin markers it is important to keep expression level low and to watch out for the formation of possible artifacts.

By contrast to Lifeact-YFP and YFP-mTn, YFP-FABD2 is not suitable as an *in vivo* marker of the tobacco pollen tube actin cytoskeleton. This fusion protein substantially inhibits tobacco pollen tube growth already at the lowest detectable expression level and therefore clearly does not allow non-invasive F-actin observation in these cells. Furthermore, as previously reported (Wilsen et al., 2006), YFP-FABD2 fails to visualize elements of the actin cytoskeleton at the pollen tube tip (subapical F-actin fringe, actin fibers connecting this structure to the F-actin network in the shank) that can readily be visualized based on low-level Lifeact-YFP or YFP-mTn expression, as well as on optimized immuno- or phalloidin labeling. Consistent with this finding, fluorescent Lifeact-fusion proteins were also reported to label fine F-actin structures at the apex of tip-growing Arabidopsis root hairs more effectively as compared to FABD2 based fluorescent marker (van der Honing et al., 2011; Sparks et al., 2016). Like Lifeact-YFP and YFP-mTn, YFP-FABD2 induces F-actin bundling and reorganization in tobacco pollen tubes in a dose-dependent manner. However, as compared to Lifeact-YFP and YFP-mTn, YFP-FABD2 appears to affect the pollen tube actin cytoskeleton in a different manner, presumably also because it reaches lower maximal expression levels than the other two markers. The accumulation of YFP-FABD2 in tobacco pollen tubes to higher levels may be prevented by rapid turnover or by cytotoxic effects of this marker, which possibly include the disruption of the subapical F-actin fringe and may cause cell death above a threshold expression level. Further investigation is required to determine whether YFP-FABD2 for the same reasons may also be confined to low expression levels in other cells types.

Interestingly, GFP-FABD2 fusion proteins were proposed to perform better than GFP-mTn as F-actin markers in transgenic Arabidopsis plants, based on comparing F-actin structures in different cell types labeled by stable expression of the two markers under the control of the constitutively active CaMV 35S promoter (Sheahan et al., 2004; Voigt et al., 2005). In the study by Voigt et al. (2005), GFP-mTn was found to be essentially diffusely distributed within specific types of Arabidopsis root cells, in which distinct F-actin structures were visualized by GFP-FABD2. Also in other cell types, GFP-FABD2 was reported to reveal more details of F-actin structure as compared to GFP-mTn (Sheahan et al., 2004). It is of course possible that the analyzed Arabidopsis cell types specifically expressed F-actin associated proteins, which effectively competed with GFP-mTn for binding sites on actin filaments, but did not prevent GFP-FABD2 from interacting with distinct binding sites on the same filaments. The reverse situation could potentially be responsible for the inability of YFP-FABD2 to visualize F-actin structures at the tip of tobacco pollen tubes, which are readily labeled by Lifeact-YFP and YFP-mTn. However, in the two studies discussed above, individual transgenic lines expressing either GFP-FABD2 or GFP-mTn were compared, which contained the two markers at unknown levels (Voigt et al., 2005) or displayed substantially stronger GFP-mTn expression (ca. 2×; Sheahan et al., 2004). It therefore remains unclear whether GFP-FABD2 in fact outperforms GFP-mTn in transgenic Arabidopsis plants, or whether GFP-FABD2 was perhaps expressed at a more suitable level in these plants. Wang et al. (2004) and Dyachok et al. (2014) also compared transgenic Arabidopsis lines stably expressing different F-actin markers and showed that a marker similar to GFP-FABD2 (lacking 25 amino acids at the N-terminus of the FABD2 domain; Wang et al., 2004), and a modified version of the same marker with an additional GFP attached at the C-terminus (GFP-FABD2^350-687^-GFP; Dyachok et al., 2014), visualized F-actin structures in different cell types that were not labeled by a mTn-GFP marker they had developed. This marker contained GFP attached to the C-terminus of the mTn domain (Wang et al., 2004; Dyachok et al., 2014), whereas generally employed GFP-mTn markers are constructed in the reverse orientation (e.g., this study). Effects of moving GFP to the C-terminus of the mTn F-actin binding domain were not systematically investigated and expression levels of the compared F-actin markers were not determined (Wang et al., 2004) or much higher in the analyzed mTn-GFP line (ca. 8×; Dyachok et al., 2014). Further investigation therefore appears to be required to unequivocally establish differences in the performances of GFP-FABD2 and GFP-mTn as F-actin markers in cells of transgenic Arabidopsis plants.

van der Honing et al. (2011) compared transgenic Arabidopsis plants expressing GFP-FABD2 or Lifeact-Venus fusion proteins at similar levels under the control of the CaMV 35S promoter. As discussed above, in these plants Lifeact-Venus was found to label apical F-actin structures at the tip of root hairs that were not visualized by GFP-FABD2. However, Lifeact-Venus affected F-actin dynamics in root epidermal cells, whereas GFP-FABD2 did not. Transgenic plants expressing Lifeact-Venus at lower levels, which were also investigated in this study, appeared to enable optimal F-actin visualization without effects on F-actin dynamics. These transgenic plants may represent the most suitable currently available tools to study F-actin structures and dynamics in Arabidopsis plants.

### Take Home Messages

(1) At low expression levels, Lifeact-YFP and YFP-mTn non-invasively label the same key components of the actin cytoskeleton in normally growing tobacco pollen tubes: (a) a network of primarily longitudinally oriented actin fibers in the shank, (b) a subapical F-actin fringe and, less effectively, (c) actin fibers connecting these two structures.

(2) At high expression levels, Lifeact-YFP, YFP-mTn and YFP-FABD2 inhibit tobacco pollen tube growth, stimulate actin filament bundling and induce F-actin reorganization. High-level Lifeact-YFP or YFP-mTn expression disrupts F-actin structures observed in normally growing pollen tubes (i.e., the subapical F-actin fringe) and induces the formation of structural artifacts including actin fibers in the apical cytoplasm and small F-actin rings.

(3) Lifeact-YFP is the marker of choice for non-invasive F-actin visualization in tobacco pollen tubes, as it has a somewhat lower potential than YFP-mTn to generate diffuse cytoplasmic background, to alter F-actin organization and to interfere with tip growth. YFP-FABD2 is not suitable as an F-actin marker in tobacco pollen tubes, because it affects F-actin organization and tip growth already at the lowest detectable expression levels, and fails to label parts of the actin cytoskeleton.

(4) With caution, key results of our analysis of the performance of Lifeact-YFP, YFP-mTn and YFP-FABD2 in tobacco pollen tubes can probably be extrapolated to other cell types. When expressed at high levels, these three markers, as well as presumably all other available GFP-based *in vivo* F-actin markers, are expected to affect F-actin organization and actin-dependent cellular processes in all cell types. However, at low expression levels at least Lifeact-YFP and YFP-mTn are likely to allow non-invasive F-actin visualization not only in normally growing pollen tubes, whose actin cytoskeleton is essential for cell expansion and extremely sensitive to F-actin disrupting drugs, but also in most other cell types. As in other cell types possible effects of markers on F-actin structure and functions are generally more difficult to assess than in pollen tubes, whose growth rate highly sensitively reacts to F-actin perturbation, markers should always be expressed at the lowest detectable level for F-actin imaging. Different GFP-based markers may label distinct F-actin structures in specific cell types, possibly because they compete for separate F-actin binding sites with differentially expressed endogenous actin binding proteins. To optimize F-actin visualization in a specific cell type, testing the performance of different markers may therefore be helpful. When comparing different markers, it is essential to ensure each marker is expressed at an optimal level.

## Author Contributions

AM-R acquired all data and contributed to the design of the work, to data analysis as well to the writing of the MS. BK conceived the work, was responsible for data analysis and interpretation, and wrote the final version of the MS.

## Conflict of Interest Statement

The authors declare that the research was conducted in the absence of any commercial or financial relationships that could be construed as a potential conflict of interest.

## Footnotes

1 https://www.xltoolbox.net/ng/
