# Supplementary figures and images for: Direct Comparison of the Performance of Commonly Employed In Vivo F-actin Markers (Lifeact-YFP, YFP-mTn and YFP-FABD2) in Tobacco Pollen Tubes
Source: Front Plant Sci. 2017 Aug 3;8:1349. doi: 10.3389/fpls.2017.01349 (PMC5540898; doi:10.3389/fpls.2017.01349)

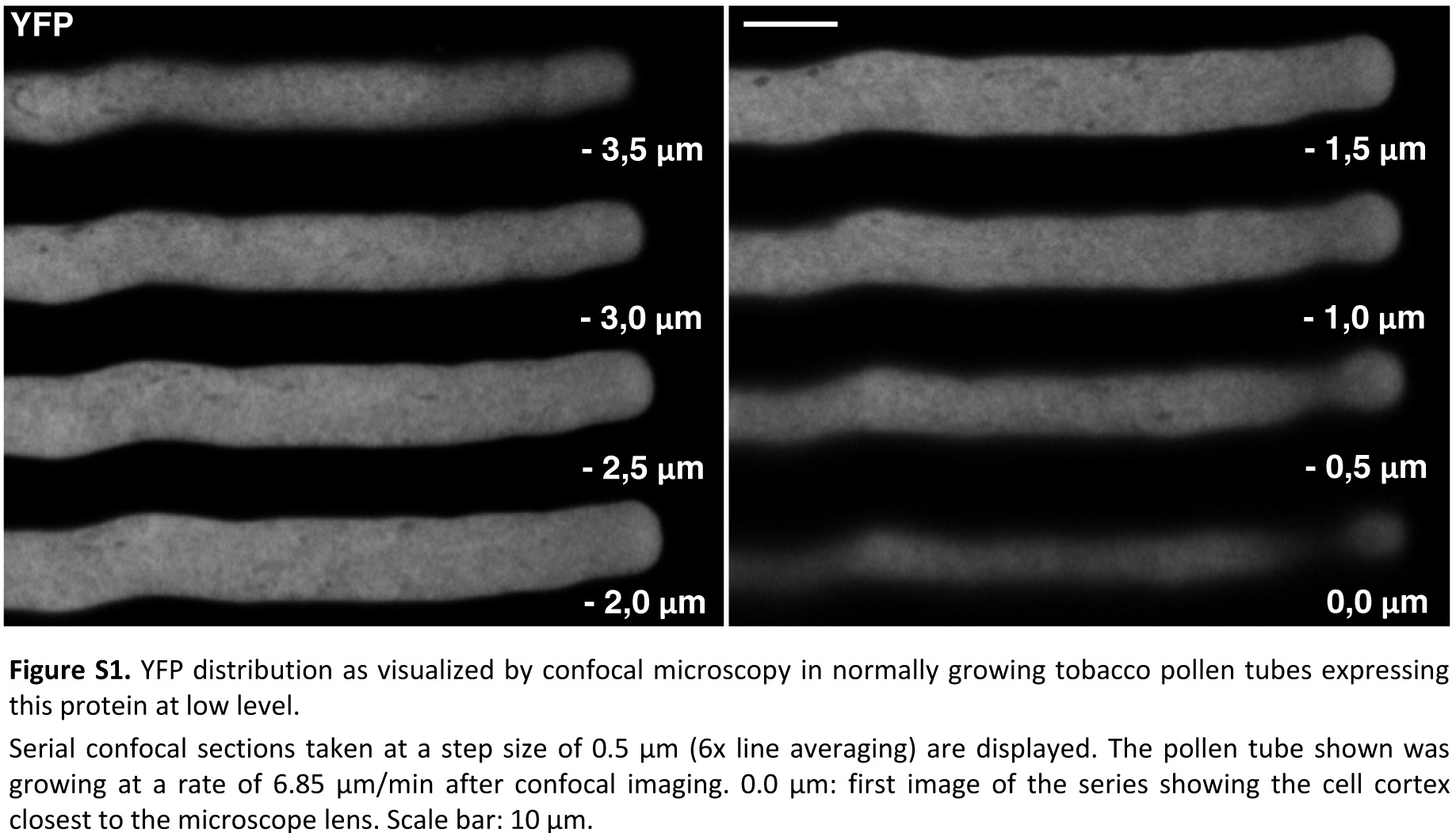

Supplement: Supplementary file 1 [file Image_1.jpeg]
